# NT-proBNP Linking Low-Moderately Impaired Renal Function and Cardiovascular Mortality in Diabetic Patients: The Population-Based Casale Monferrato Study

**DOI:** 10.1371/journal.pone.0114855

**Published:** 2014-12-11

**Authors:** Graziella Bruno, Federica Barutta, Andrea Landi, Paolo Cavallo Perin, Gabriella Gruden

**Affiliations:** Department of Medical Sciences, University of Turin, Turin, Italy; University of Perugia, Italy

## Abstract

**Background:**

Few data are available to assess whether a low-moderate reduction in estimated glomerular filtration rates (eGFR) has a role per se on cardiovascular (CV) mortality or other biomarkers such as NT-proBNP allow to explain such association.

**Methods and Findings:**

In a prospective study including 1,645 type 2 diabetic subjects of the population-based Casale Monferrato Study, who had no clinical evidence of heart failure and eGFR >45 ml/min/1.73 m^2^, we examined 6 years CV mortality. Multivariate Cox proportional hazards modeling were used to estimate the effect of NT-proBNP on the association between eGFR and mortality, independently of baseline CV risk factors, albumin excretion rate (AER) and C-reactive protein (CRP). During follow-up, 327 people died (149 of CV diseases) out of 8334.5 person-years. Compared to eGFR≥90 ml/min/1.73 m^2^, values of 60–89 and 45–59 ml/min/1.73 m^2^ conferred a fully adjusted hazard ratios (HRs) of CV mortality of 1.74 (1.08–2.82) and 1.95 (1.03–3.68), respectively. After further adjustment for NT-proBNP, however, HRs were no longer significant (HRs 1.42, 0.83–2.42 and 1.22, 0.59–2.51). In this model, HR for logNT-proBNP was 1.84 (1.52–2.22). Adding NT-proBNP to the model improved the C-statistic of CV mortality from 0.79 (0.76–0.83) to 0.84 (0.81–0.87), yielded an IDI of 0.03 (p = 0.02), and a NRI of 0.44 (p = 0.016).

**Conclusions:**

In diabetic people a modest reduction in renal function increased 6-year CV mortality independently of albuminuria. This association, however, was mainly explained by the effect of NT-proBNP, that remained the strongest prognostic marker for a worse CV outcome, even after adjustment for other CV risk factors and pre-existing CVD.

## Introduction

Diabetes is a leading cause of chronic kidney disease (CKD) in the Western countries and patients with CKD are exposed to increased morbidity and mortality as a result of cardiovascular (CV) events [1]. Early detection of CKD by estimating glomerular filtration rate (eGFR) using creatinine-based formulas has been suggested as a valid tool to improve patients stratification and to intensify treatment in subgroups of diabetic patients at highest risk of CV events [2]. In addition, a synergistic relationship between eGFR and microalbuminuria in predicting long-term progression to end-stage renal disease (ESRD) and CV death has been recently confirmed in large cohorts of diabetic patients [3], emphasizing the importance of their combined assessment to improve risk prediction.

Despite the growing recognition of the relationships between kidney and CV diseases along the cardiorenal continuum, the underlying pathophysiology remains poorly understood. Endothelial dysfunction, cardiac remodelling, and atherosclerosis occurs in early CKD stages and are believed to contribute to the enhanced CV risk of patients with CKD [4]. Discovery of novel biomarkers, mirroring these early pathogenetic events and possibly mediating the epidemiological relationship between CKD and CV events, may significantly improve risk stratification, particularly in the subgroup of type 2 diabetic patients at low-moderate risk [5].

Human natriuretic peptides genes encode for long inactive peptides that are processed to active peptides and equimolar concentrations of inactive fragments. PreproBNP is converted in proBNP by removal of a 26-amino acid sequence. Pro-BNP, which is often post-translationally O-glycosylated in the N-terminal region, is cleaved by furin into BNP and the inactive fragment NT-proBNP [6]. Compared to BNP, NT-proBNP has a longer plasma half-life, making it a more suitable biomarker for clinical use. Cardiac stretch causes secretion of BNP (32aminoacid-long peptide hormone) and NT-proBNP (76 aminoacid-long peptide) which are released in equimolar amounts into the circulation and are physiological antagonists of the renin-angiotensin system. The N-amino terminal fragment of the prohormone B-type natriuretic peptide (NT-proBNP) is released from cardiomyocytes in response to ventricular wall stretch/tension and is a sensitive marker of both left ventricular hypertrophy and volume expansion [6]. We have recently reported that NT-proBNP is also a strong independent predictor of short-term CV mortality risk in type 2 diabetic patients, including those without preexisting CVD [7]. NT-proBNP values are enhanced in subjects with CKD [8], but the clinical interpretation of these results in asymptomatic patients has been a source of controversy [9]. From a clinical point of view, it is of particular relevance the identification of CV predictors in the subgroup of patients with only moderately reduced renal function. However, prospective data on the relationship between eGFR, albuminuria, and NT-proBNP on CV mortality in these patients are limited [10] and no data are available in type 2 diabetic patients.

In the present study, we have prospectively assessed the effect of NT-proBNP on the association of moderately reduced eGFR levels with 6-years CV mortality, independently of classical and new cardiovascular risk factors, including albuminuria and plasma C-reactive protein (CRP), in a population-based cohort of people with type 2 diabetes and eGFR >45 ml/min/1.73 m^2.^ [11]–[12].

## Subjects and Methods

### Baseline Study

The Casale Monferrato survey was begun in 1988 with the aim of assessing the prevalence of known diabetes in persons living in the area of Casale Monferrato, Northern Italy (93477 inhabitants). The study design has been described in detail elsewhere [11]–[13]. Briefly, persons with a previous diagnosis of diabetes (n = 1967) were identified from diabetes clinics, general practitioners, hospital discharge records, prescriptions and records of sales and reagent strips and syringes. The completeness of ascertainment (80%) was estimated by applying log-linear models to the capture-recapture methods, making it possible to take into account both dependence between data sources and the heterogeneity of patients within sources [11]. In 2000 a new survey included all the members of the original cohort who were still alive and living in Casale Monferrato (N = 860), and in addition all the persons with a new diagnosis of type 2 diabetes (N = 2389), with an estimated completeness of ascertainment of 95%. All the subjects are white. Of the 3,249 type 2 diabetic subjects enrolled in the Casale Monferrato Study, we excluded 1463 patients who had no samples available for NT-proBNP, 56 patients who had clinical evidence of acute and or chronic heart failure, and 85 patients with eGFR <45 ml/min/1.73 m^2^, leading to a final cohort of 1,645 (50.6%) patients recruited for present analyses.

The date of diabetes diagnosis was retrieved and recorded for all recruited subjects. As described in detail elsewhere, patients were interviewed, examined, and blood samples collected after overnight fasting [12]. Ethics approval was obtained by the Alessandria Hospital Ethical Committee and all subjects provided written informed consent. Blood samples were collected at the baseline examination in 2000 in 60% of the cohort, depending on their consent to provide further blood samples for future research purposes. Smoking status was classified as current smoker, never smoker, ex-smoker (smoking cessation at least a month prior to the visit). Hypertension was defined as systolic blood pressure >140 mmHg and/or diastolic blood pressure >90 mmHg or treatment with antihypertensive drugs. CVD was defined as a positive medical history of a CV event, including myocardial infarction, angina pectoris, coronary artery bypass graft and stroke, and/or ischemic changes on a resting 12-lead electrocardiogram, classified according to the Minnesota Code. The WHO Rose questionnaire was also administered and people with symptoms suggestive of CVD underwent further investigations to confirm the diagnosis. Congestive heart failure was clinically diagnosed based on medical history, physical examination, and drug treatment and were considered an exclusion criteria for this analysis.

Blood samples were analysed at the Central Laboratory [12]. Triglycerides, total cholesterol, high-density lipoprotein (HDL) cholesterol, apolipoprotein A1 (apoA1), apoB, serum creatinine, and hemoglobin A1c (HbA1c) (reference range, 3.8–5.5%) were measured by standard laboratory techniques. Low-density lipoprotein (LDL) cholesterol was calculated from Friedewald's formula when triglyceride values were <4.48 mmol/l. Glomerular filtration rate (GFR) was estimated using the four component abbreviated equation from the MDRD Study [14]. High-sensitivity (hs)-CRP was measured by immunoturbidimetry (Roche-Diagnostic, coefficient of variation  = 0.5%). Albumin excretion rate (AER) was measured by nephelometry (Behring Nephelometer Analyzer, Behring Institute, Marburg, Germany, coefficient of variation: 4%) on single overnight urine collections and categorised as either normoalbuminuria (<20 µg/min) or micro/macroalbuminuria (≥20 µg/min). Serum NT-proBNP levels were measured by a two-site sandwich electrochemiluminescence immunoassay (Elecsys proBNP II, Roche Diagnostic, Mannheim, Germany), using a Modular Analytics Evo analyzer with a E170 module (Roche) as previously described [15]. The intra-assay variation was below 3.0% and total coefficient of variation ranges between 2.2 and 5.8% in low and high ranges of NT-proBNP.

### Follow-Up Study

Six years after the baseline examinations, mortality data up to December 31^st^, 2006 were obtained from the demographical files of towns of residence and both hospital and autopsy records. Only one patient was lost to follow-up. Underlying causes of death were derived and coded by two authors, according to the 9^th^ revision of the International Classification of Disease (ICD). Cardiovascular mortality was defined by ICD-9 codes 390–459.

### Statistical Analysis

Variables distributed normally are presented as mean and standard deviation (SD), whereas variables with skewed distribution were analysed after natural logarithmic transformation (triglycerides, NT-proBNP, CRP) and results presented as geometric means. Anova test with Scheffe method for multiple comparisons was employed to compare features of people across levels of eGFR. Mortality rates were calculated dividing the number of deaths, occurring during the study period, by the number of person-years of observation. Cox proportional hazards modelling was used to estimate the hazard ratios (HRs) and 95% confidence intervals (95% CI) of cardiovascular and all-cause mortality by eGFR values, independently of conventional and new risk factors. Analyses were performed using strata of eGFR. In order to assess the role of NT-proBNP on the association of eGFR with respect to mortality, all models were adjusted for age, sex, diabetes duration (model 1), hypertension, HbA1c, logCRP, smoking, LDD-cholesterol, logAER, CVD (model 2) and logNT-proBNP (model 3). Models were constructed with variables as continuous measures to provide maximum power for detecting an association between eGFR and mortality. In addition, we allowed any of the following variables to enter the model if they add significantly or modified HRs of NT-proBNP: antihyperglycemic treatment (diet, oral drugs, insulin), treatment with ACE/ARB inhibitors, diuretics and other antihypertensive treatment, statins, BMI, waist circumference, uric acid, total, HDL-cholesterol, triglycerides, fibrinogen, systolic, diastolic and pulse blood pressure. We tested for linear trends across eGFR strata by entering a single ordinal term into the Cox regression model. The proportional hazard assumptions of explanatory variables were assessed on the basis of Schoenfeld residuals. The likelihood ratio (LR) test was used to assess the statistical significance of examined variables in nested models. A P value of less than 0.05 was considered to indicate statistical significance. We also assessed discrimination, that is the ability to separate diabetic people of the Casale Monferrato cohort who will die and those who will survive during the 6-years follow-up period, through C-statistic, which can range from 0.5 (no predictive ability) to 1 (perfect discrimination). In our analysis, the C statistic shows the proportion of pairs for which the model assigns higher probability to the person who has died than to the person who has survived. We additionally investigated the integrated discrimination improvement (IDI). Reclassification of participants was assessed by categorical net reclassification improvement (NRI) into risk categories of the cumulative risk of cardiovascular death (<33%, 33–66% and>66%) [16]. Analyses were performed with STATA software, version 10.0.

## Results

As compared to the all cohort, patients included in the present study were younger (67.8±10.4 vs 70.6±12.2 years, p<0.0001), had slight lower diabetes duration (10.8±7.9 vs 11.3±8.4 years, p = 0.02) and negligible lower HbA1c values (7.0%±1.8 vs 7.2%±1.7, 53 mmol/mol ±19.7 vs 55±19.7, p = 0.05), but similar systolic (146.0±16.1 vs 145.2±17.5 mmHg, p = 0.28) and diastolic (82.7±8.2 vs 83.1±8.2 mmHg, p = 0.18) blood pressure levels. At the baseline examination, most of subjects (64.5%) were elderly, being 65 years and over at recruitment.

The baseline features of the cohort by categories of eGFR are shown in Table 1. Log-NT-proBNP and eGFR values were negatively correlated (r = −0.28, p<0.0001).

**Table 1:** Characteristics of people with type 2 diabetes in the Casale Monferrato Study with NT-proBNP assessment at the baseline examination (2000), by estimated glomerular filtration rate (eGFR)

	eGFR ml/min/1.73 m^2^			
	≥90 (n = 716)	60–89 (n = 777)	45–59 (n = 152)	p value
**Age (years)**	63.3±10.6	69.9±9.0	74.0±8.5	<0.001
**Males (%)**	385 (53.8%)	378 (48.6%)	55 (36.2%)	<0.001
**Duration of diabetes (years)**	9.4±7.3	11.1±7.9	12.8±8.5	<0.001
**BMI (kg/m^2^)**	29.2±5.5	28.3±4.8	28.6±5.5	0.02
**HbA1c (%)**	7.1±1.8	6.8±1.7	7.2±1.8	<0.001
**CRP** a **(mg/l)**	2.7 (1.2–6.0)	2.7 (1.2–5.7)	3.5 (1.6–8.0)	<0.001
**Total Cholesterol (mmol/l)**	5.44±1.05	5.56±1.02	5.55±1.01	0.09
**LDL-cholesterol (mmol/l)**	3.28±0.87	3.37±0.89	3.32±0.86	0.04
**HDL-cholesterol (mmol/l)**	1.41±0.38	1.45±0.40	1.44±0.37	0.01
**Triglycerides (mmol/l)** a	1.42	1.43	1.57	<0.001
**ApoA1 (mg/dl)**	155.3±38.0	157.0±38.2	158.4±36.3	0.06
**ApoB (mg/dl)**	101.5±26.7	102.8±26.2	107.5±29.0	0.11
**ApoB/apoA1**	0.71±0.32	0.71±0.31	0.73±0.35	0.41
**Fibrinogen (g/l)**	3.7±0.7	3.8±0.7	4.0±0.8	0.001
**Uric acid (µmol/l)**	297.40±77.32	344.98±118.96	130.67±83.27	<0.001
**Systolic blood pressure (mmHg)**	144.6±16.0	146.2±15.6	149.1±17.6	<0.001
**Diastolic blood pressure (mmHg)**	82.5±8.4	82.6±7.6	83.5±8.6	0.73
**Hypertension (%)**	610 (85.4%)	713 (91.8%)	145 (95.4%)	<0.001
**CVD (%)**	124 (17.3%)	175 (22.5%)	44 (29.0%)	<0.001
**AER (µg/min) 20–200**	166 (27.7%)	151 (23.5%)	33 (27.3%)	<0.001
**>200**	19 (3.2)	33 (5.1%)	14 (11.6%)	
**NT-proBNP (pg/ml)** a	56.0 (25–120)	102.3 (48–221)	182.3 (94–347)	<0.001

a geometric mean and interquartile range.

During the 6-years follow-up period, 327 people died out of 8334.5 person-years. Numbers of deaths for decreasing eGFR stratum were 111, 176, and 40, respectively. CV mortality accounted for 149 (45.6%) deaths and out of them 90 deaths occurred in subjects without pre-existing CVD.

Compared to people with eGFR ≥90 ml/min/1.73 m^2^, those with eGFR of 60–89 ml/min/1.73 m^2^ had HR for CV mortality of 1.31 (0.99–1.88) and those with values of 45–59 ml/min/1.73 m^2^ HR of 1.86 (1.23–2.83), independently of age, sex, and diabetes duration (Table 2, model 1). Further adjustment for other risk factors, slightly modified HRs (model 2). After inclusion of logNT-proBNB into the fully adjusted model (model 3), however, HRs of eGFR decreased to non significant values, whereas HR for logNT-proBNP was 1.84 (1.52–2.22). Results were similar including in models eGFR as continuous rather than categorical variable. No changes in HRs were observed after the inclusion into the model of BMI, antidiabetic treatments, pulse pressure and treatment with ACE/ARB, diuretics, statins.

**Table 2:** Results of Cox-regression analysis of variables associated with 6-years all-cause and cardiovascular mortality in people with type 2 diabetes of the Casale Monferrato Study.

	Model 1	Model 2	Model 3
***Cardiovascular mortality***			
	**HR (95% CI)**	**HR (95% CI)**	**HR (95% CI)**
**eGFR ml/min/1.73 m^2^**			
90+	1.00	1.00	1.00
60–89	1.31 (0.99–1.80)	1.74 (1.08–2.82)	1.42 (0.83–2.42)
45–59	1.86 (1.23–2.83)	1.95 (1.03–3.68)	1.22 (0.59–2.51)
*p value for trend*	*<0.001*	*0.02*	*0.49*
**logNT-proBNP**			1.84 (1.52–2.22)
***All-cause mortality***			
	**HR (95% CI)**	**HR (95% CI)**	**HR (95% CI)**
**eGFR ml/min/1.73 m^2^**			
90+	1.00	1.00	1.00
60–89	1.08 (0.88–1.33)	1.26 (0.95–1.67)	1.09 (0.79–1.50)
45–59	1.36 (1.01–1.83)	1.19 (0.78–1.82)	0.93 (0.50–1.49)
*p value for trend*	*0.06*	*0.22*	*0.94*
**logNT-proBNP**			1.51 (1.33–1.70)

Data are shown as hazard ratios (HRs) and 95% confidence intervals (95% CI).

HRs were independent of each other.

Model 1: adjusted for age, sex and diabetes duration;

Model 2: adjusted for model 1 adjustments plus HbA1c, LDL-cholesterol, hypertension, smoke, logCRP, logAER, CVD

Model 3: adjusted for model 2 adjustments plus logNT-proBNP.

As regards to all-cause mortality, HRs of eGFR were not significant in all models, whereas logNT-proBNP was significant even in the fully adjusted model 3.

We then examined the discrimination effect of adding NT-proBNP measurement to eGFR, classical cardiovascular risk factors and AER, comparing models with and without NT-proBNP. The C-statistic pointed out a small advantage in using NT-proBNP for cardiovascular mortality: 0.79 (95% CI 0.76–0.83), and 0.83, (95% CI 0.81–0.87) without and with NT-proBNP, respectively. The NRI and the IDI were 0.44 (p = 0.016) and 0.03 (p = 0.02), respectively.

## Discussion

Our prospective analysis of the population-based Casale Monferrato Study provides evidence that, in type 2 diabetic patients with moderately reduced renal function, the enhanced risk of CV mortality associated with eGFR is mainly explained by increased plasma values of NT-proBNP, which were strongly associated with mortality, even after multiple adjustments, including AER and CRP. Indeed, when we analysed in a multivariate model the contribution of eGFR and NT-proBNP, independently of each other and of other CV risk factors, we provided evidence that NT-proBNP values were strongly associated with CV mortality, whereas the well-known association with eGFR disappeared [17].

This finding indicate that NT-proBNP is either a confounder or a risk factor explaining the association between eGFR and mortality in these patients. The interpretation of increased values of NT-proBNP in asyntomatic people with reduced eGFR is a matter of controversy, as in patients with CKD NT-proBNP are typically elevated and few prognostic data are available. However, in the present report we point out that increased plasma NT-proBNP values have a role per se, which do not reflect diminished renal clearance of natriuretic peptides. The prognostic impact of NT-proBNP is preserved even in diabetic patients with eGFR 60–89 ml/min/1.73 m^2^ compared to those with higher values, and appear to fully explain the association between eGFR and CV mortality. Indeed, in a fully adjusted model, the statistical significance of HR for eGFR disappeared, whereas HR for NTproBNP was not modified. It is likely that NT-proBNP integrate the information provided by several other risk factors associated with moderate reduction of renal failure, such as arterial stiffness and volume overload, allowing a better definition of individual risk. Although NT-proBNP is inactive, it is secreted in equimolar concentrations with BNP, which, as recently reviewed [6], has emerged as key mediators in the control of metabolic processes, including the heart in the network of organs that regulate energy usage and metabolism. Epidemiological studies have shown that natriuretic peptides are reduced in people with obesity, insulin-resistance, diabetes and this deficiency may contribute to enhance their global cardiovascular risk [6]. Therefore, it is now evident that BNP/NT-proBNP can provide quantitative information about the state of cardiovascular health across the all spectrum of CVD, with relevant clinical implications.

Our findings are consistent and expand results from the few studies examining the intertwined effects of NT-proBNP, renal dysfunction, and albuminuria on CV mortality. The PREVEND study, performed on a large cohort of non-diabetic subjects, has shown that NT-pro-BNP levels were associated with CV both incident events and mortality even after adjustment for eGFR and albuminuria [10]. However, NT-proBNP cut-off value was greater than in our study. A recent prospective cohort study nested within the Trial to Reduce Cardiovascular Events With Aranesp Therapy (TREAT) has reported that troponin T and NT-proBNP enhance prediction of ESRD outcomes, including death, beyond established risk factors, eGFR, and proteinuria in type 2 diabetic patients with CKD and anaemia [18]. However, the study was nested within a randomized clinical trial, median NT-proBNP were relatively elevated due to the more advanced stage of CKD of recruited patients, and no separate analysis was performed for CV mortality that was under-represented. Therefore, our study is the first to assess the prognostic value of NT-proBNP for CV mortality in relation to eGFR and albuminuria in a large population-based cohort of type 2 diabetic patients.

In subjects with decreased renal function the use of NT-proBNP has been debated on the assumption that NT-proBNP levels may falsely increase because of reduced renal NT-proBNP clearance with advancing CKD [9]. However, it has been shown that renal fractional extraction for NT-proBNP diminishes only modestly across a range of eGFRs and correlates minimally with eGFR [9]. Our data on the superior prognostic impact of moderately elevated NT-proBNP values with respect to eGFR values in diabetic patients with moderately impaired renal function would support the clinical usefulness in routine practice of this marker. We have recently suggested that NT-proBNP might have a relevant role in early identification of type 2 diabetic patients at increased CV risk as in the Casale Monferrato cohort a slight increase in NT-proBNP levels was a strong independent predictor of CV mortality even in patients without pre-existing CVD [7]. The present study strengthens this argument by showing that NT-proBNP also explain at least in part the predictive effect of eGFR on CV mortality. The underlying pathophysiology remains unclear; however, natriuretic peptides and NT-proBNP are likely to directly reflect the subclinical cardiac damage from silent myocardial ischemia, left ventricular hypertrophy, or increased apoptosis that impacts on CV mortality and is often associated with moderately reduced renal function [6].

Strengths of this study are the prospective design; the recruitment of a large population-based cohort of type 2 diabetic people, increasing both precision and generalizability; the centralized assessment of CV risk factors and biomarkers, allowing to analyze the potential confounding effect of covariates; the high degree of completeness of follow-up data. There are, however, certain limitations. Only 50.6% of patients of the original population-based cohort were recruited in the study and they were younger than unrecruited people. As age was positively correlated with NT-proBNP, the association between eGFR and mortality might have been even greater in the entire cohort. Our results are based on an observational prospective cohort and, though multivariate methods of analyses were used to control for the effect of known confounders in the relationship between NT-proBNP and mortality, we cannot rule out the possibility of residual or undetected confounding effects on our results. A single NT-proBNP measurement was performed using stored samples collected at baseline. Although the stability of NT-proBNP after long term storage is unknown, all samples were treated and stored under the same conditions. The study is based on a cohort of mainly elderly subjects and, though this is a group of considerable clinical interest because they constitute a high-risk group in whom traditional risk factors become less predictive [19], our results need further confirmation in similar middle-aged populations. Finally, no data on structural and functional cardiac abnormalities were available.

In conclusion, this population-based study shows that in people with type 2 diabetes a moderate reduction in renal function increased 6-year CV mortality independently of albuminuria. The association between eGFR and mortality, however, was explained by the effect of NT-proBNP, which was a stronger independent predictor of CV mortality, even in the earlier stage of CKD. Further studies are warranted to assess if combining AER with NT-proBNP rather than with eGFR may improve CV risk stratification in type 2 diabetic patients as well as in the general population.

## Supporting Information

S1 Data File of baseline data.
(DTA)
